# Isolated Distal Ulnar Salter-Harris IV Fracture in an Adolescent Male With Distal Radioulnar Joint Injury: A Case Report and Review of the Literature

**DOI:** 10.7759/cureus.107637

**Published:** 2026-04-24

**Authors:** Pranav Krish, Robert D Faccone, Matthew Heffelfinger, Daniel Acampa, Baruch Toledano

**Affiliations:** 1 Osteopathic Medicine, New York Institute of Technology, Old Westbury, USA; 2 Orthopedic Surgery, Nassau University Medical Center, East Meadow, USA

**Keywords:** distal radioulnar joint, distal ulna fracture, druj instability, growth plate injury, kirschner wire fixation, pediatric physeal injury, salter harris iv fracture, shiv, wrist trauma

## Abstract

Distal ulnar physeal fractures are uncommon in pediatric patients, and isolated Salter-Harris IV (SHIV) injuries of the distal ulna are particularly rare. These injuries may raise concern for distal radioulnar joint (DRUJ) incongruity, limited forearm rotation, and future growth disturbance. Management strategies vary depending on fracture displacement, reducibility, and joint stability.

A 14-year-old male patient presented with left wrist pain three days after a bicycle accident. Examination demonstrated tenderness over the distal ulna and restricted wrist and forearm motion, including pronation and supination. Radiographs showed a SHIV fracture of the distal ulna. On presentation, the patient underwent closed reduction and sugar-tong splint immobilization in the emergency department. After closed reduction, forearm rotation remained limited, and concern persisted for DRUJ incongruity/mechanical block, prompting operative fixation the following day. Open reduction and Kirschner wire fixation were performed through a dorsal approach with fluoroscopic confirmation of fracture alignment and DRUJ congruity. Follow-up radiographs at approximately two weeks and six weeks demonstrated maintained alignment and hardware position. The patient did not return for additional follow-up, limiting long-term assessment for physeal arrest, DRUJ symptoms, and functional outcome.

This case describes a rare distal ulnar SHIV fracture in an adolescent and highlights the importance of individualized management based on fracture characteristics and DRUJ assessment. In this patient, surgery was selected because of concern for rotational block/instability rather than as a general recommendation for all distal ulnar physeal injuries. Longitudinal follow-up remains important in these injuries because growth-related complications may not be clinically apparent early after treatment.

## Introduction

Distal forearm fractures are common in pediatrics, with more than one out of every four pediatric fractures involving the distal radius [1,2]. Distal ulnar physeal fractures occur less frequently, and isolated distal ulnar Salter-Harris IV (SHIV) fractures are rare, occurring in only 9.2% of all physeal fractures [3,4]. A SHIV injury extends through the metaphysis, physis, and epiphysis, raising concern for both articular incongruity and physeal disturbance [1-4]. These injuries may also involve the distal radioulnar joint (DRUJ), the articulation between the distal radius and ulna that is essential for forearm pronation and supination [1-4]. 

Although uncommon, injuries involving the distal ulnar physis may be clinically important because of the distal ulna’s contribution to longitudinal growth and the functional role of the DRUJ in forearm rotation [4]. Reported concerns after distal ulnar physeal injury include premature physeal closure, angular deformity, ulnar variance changes, pain, and limitation in forearm rotation [4]. At the same time, management is not uniform. Depending on fracture displacement, reducibility, joint congruity, and symptoms, treatment may range from immobilization to operative fixation [4,5]. Isolated distal ulnar SHIV injuries are particularly infrequent, and the associated concern for DRUJ mechanics can complicate decision-making [4,6].

This is a case of an isolated distal ulnar SHIV fracture in a 14-year-old male patient. The case is presented to highlight clinical assessment, operative decision-making in the setting of concern for DRUJ mechanical block/instability, and the importance of long-term follow-up when physeal injury is involved.

## Case presentation

Initial presentation and management

A 14-year-old male patient presented to the emergency department on 5/14/2024 with a complaint of pain in the left wrist after a bicycle accident three days prior. The patient complained of pain at a 6/10 severity, and described it as constant and aching. The pain was exacerbated by motion and palpation but alleviated with rest and immobilization. The patient was right-hand dominant and denied numbness, tingling, weakness, or any other orthopedic complaints.

Physical examination revealed intact skin with no overlying bony deformities. Significant findings included limitations in range of motion (ROM) in left wrist flexion, extension, pronation, and supination, with tenderness to palpation over the distal ulna. Neurovascular examination was intact distally. The limitation in forearm rotation raised concern for possible DRUJ involvement.

**Figure 1:**
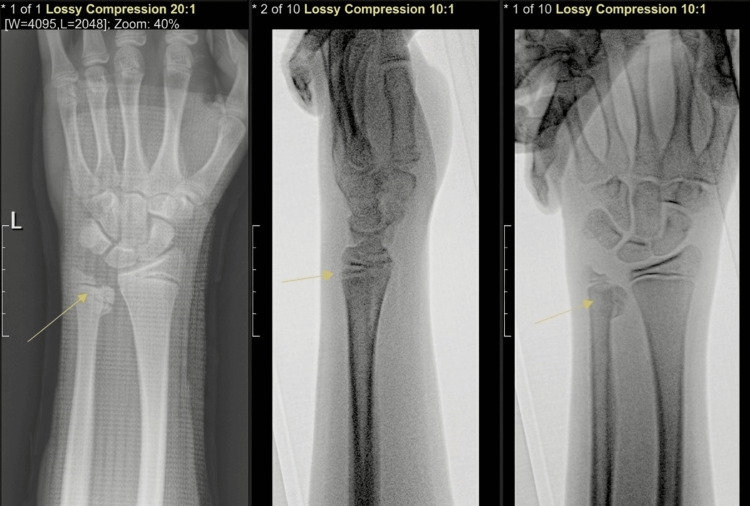
Radiographs obtained at the initial emergency department presentation Preoperative radiographs of the left wrist anteroposterior/posteroanterior (AP/PA; left panel), lateral (middle panel), and oblique (right panel), demonstrating a distal ulnar physeal fracture consistent with a Salter Harris IV (SHIV) fracture pattern (Day 0).

Anteroposterior (AP), lateral, and oblique radiographs of the extremity revealed a SHIV fracture of the left distal ulna (Figure 1).

On presentation (5/14/2024), the patient underwent closed reduction and sugar-tong splint immobilization in the emergency department. Given that forearm rotation remained limited and concern persisted for DRUJ incongruity/mechanical block, the patient was scheduled for operative fixation the following day (5/15/2024).

**Table 1:** Clinical timeline of case presentation, management, and follow-up ROM: range of motion; AP: anteroposterior; DRUJ: distal radioulnar joint; ORIF: open reduction and internal fixation; ED: emergency room; AP: anteroposterior.

Date	Time from ED evaluation	Encounter/Intervention	Key findings
5/11/24	Day -3	Injury	Bicycle accident with left wrist pain onset
5/14/24	Day 0	Emergency department evaluation	Radiographs demonstrated distal ulnar physeal fracture consistent with Salter-Harris IV; exam notable for limited wrist ROM and restricted pronation/supination
5/14/24	Day 0	Initial management	Closed reduction and sugar-tong splint immobilization performed in the ED
5/15/24	Day 1	Operative management	ORIF via dorsal approach with 1.25-mm K-wire fixation; second K-wire placed across the DRUJ into the distal radius to maintain joint reduction
5/28/24	Day 14 (~2 weeks)	Follow-up imaging	AP/lateral/oblique radiographs showed stable hardware position and no obvious interval loss of alignment on available views
6/28/24	Day 45 (~6 weeks)	Follow-up imaging	AP/lateral/oblique radiographs showed stable hardware position and maintained alignment on available views

Operative technique

Under general anesthesia, fluoroscopy was used to localize the fracture. A dorsal incision was made along the ulnar aspect of the DRUJ, and dissection was carried down to the extensor retinaculum. The retinaculum was incised between the fourth and fifth compartments, and a dorsal capsulotomy was performed to expose the fracture. The ulnar styloid fragment was identified as dorsally displaced relative to the shaft.

**Figure 2:**
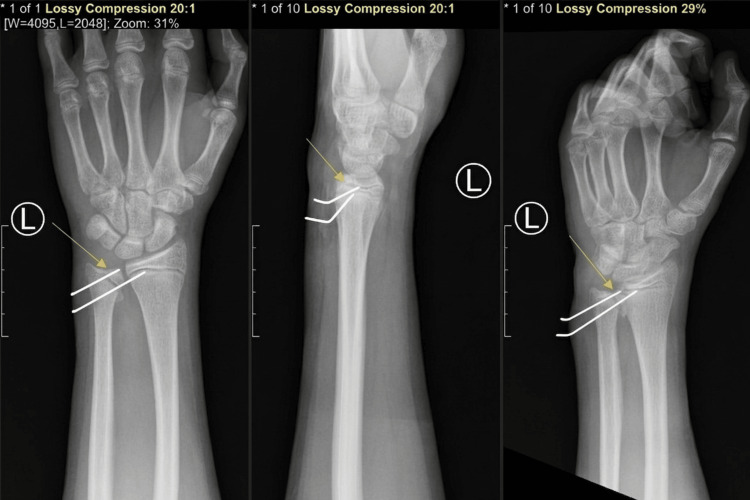
Radiographs obtained immediately after the surgery Postoperative radiographs of the left wrist anteroposterior/posteroanterior (AP/PA; left panel), lateral (middle panel), and oblique (right panel), demonstrating Kirschner wire fixation of the distal ulna (Day one).

After evacuation of the hematoma and irrigation, the fracture was reduced using a Freer elevator (Aesculap, B. Braun Company, Germany) and held with pointed reduction forceps. Fluoroscopy confirmed improved alignment. Two 1.25-mm Kirschner wires (Synthes GmbH, Oberdorf, Switzerland) were then placed: one across the fracture from the shaft into the distal fragment and a second transfixing the DRUJ into the distal radius to maintain DRUJ alignment. Final fluoroscopic imaging in AP, lateral and oblique views demonstrated satisfactory fracture alignment, hardware position, and DRUJ alignment (Figure 2).

The capsule was repaired, the skin was closed, and the patient was immobilized in a long arm cast with the elbow flexed at 90 degrees and the forearm in neutral. The patient was discharged from the post-anesthesia care unit in stable condition. 

Follow-up

**Figure 3:**
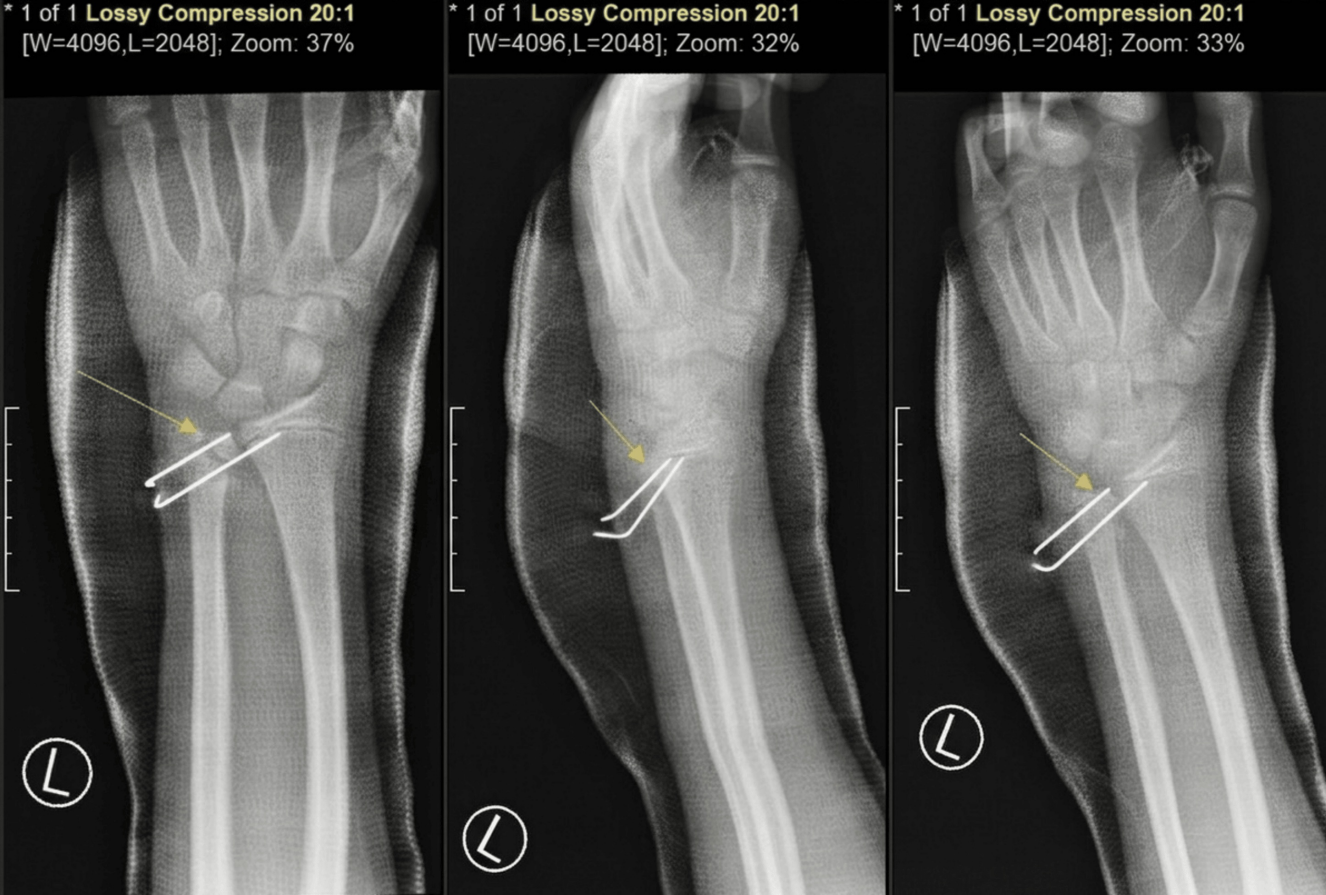
Radiographs at the two-week postoperative follow-up Radiographs of left wrist anteroposterior/posteroanterior (AP/PA; left panel), lateral (middle panel), and oblique (right panel), demonstrating stable Kirschner wire position and no obvious interval loss of alignment on the available view (Day 14).

**Figure 4:**
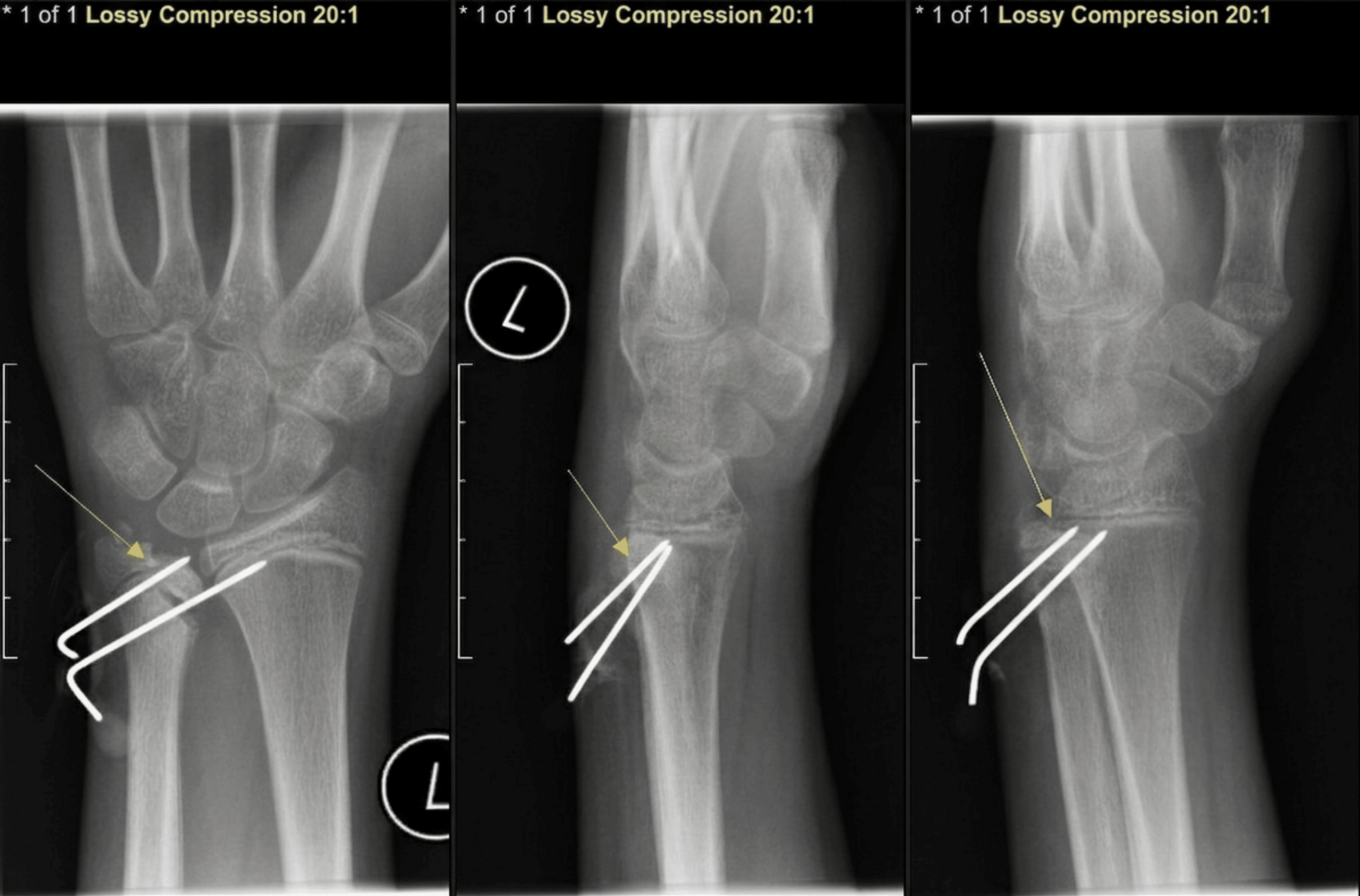
Radiographs at ~six weeks postoperative follow-up Radiographs of the left wrist anteroposterior/posteroanterior (AP/PA; left panel), lateral (middle panel), and oblique (right panel), demonstrating stable Kirschner wire position and maintained alignment (Day 45).

AP, lateral and oblique radiographs obtained at two weeks postoperatively and again at six weeks (Figures 3, 4) showed maintained alignment and stable hardware position on available imaging, without obvious interval loss of reduction.

The patient did not return for subsequent follow-up appointments. As a result, long-term assessment for physeal arrest, growth disturbance, DRUJ symptoms, and functional recovery was not available.

## Discussion

Isolated distal ulnar physeal fractures are uncommon in pediatric wrist trauma, and isolated distal ulnar SHIV injuries are particularly rare [4,6]. As a result, most available evidence consists of case reports and small retrospective series, which limits consensus on a single “best” treatment strategy [4-8]. Across the literature, management decisions are typically individualized based on fracture morphology (especially intra-articular involvement), the ability to obtain and maintain an acceptable reduction, and clinical assessment of the DRUJ congruity and forearm rotation [4,6-10].

Although distal ulnar physeal injuries are less common than distal radius fractures, they are clinically important because the distal ulna contributes substantially to ulnar longitudinal growth and because the DRUJ is critical for pronation and supination [4,6,11-13]. Prior studies and reviews describe potential sequelae after distal ulnar physeal injury including premature physeal closure, progressive ulnar variance change, angular deformity, wrist pain, decreased forearm rotation, and DRUJ symptoms [4,11-15]. Importantly, these complications may not be clinically apparent early after injury or early after reduction, reinforcing the value of longitudinal clinical and radiographic surveillance [4,6,12-15].

The existing literature supports a spectrum of initial management. Many distal ulnar physeal fractures can be treated nonoperatively with immobilization, with or without closed reduction, particularly when radiographs demonstrate <2 mm displacement, minimal angulation, preserved DRUJ congruity, and when examination shows no gross DRUJ instability or persistent subluxation [4,6,8-10,16]. However, operative fixation has been reported and may be considered in selected circumstances, including irreducible fractures, persistent displacement, intra-articular incongruity (as in SHIV injuries), and cases with suspected or confirmed DRUJ instability or a mechanical limitation to forearm rotation [4,7-11,17]. Since open reduction and transphyseal fixation may increase risk of physeal insult, operative intervention is generally reserved for irreducible fractures, unacceptable intra-articular incongruity, or persistent DRUJ instability/mechanical block [4-8,17]. Published reviews, therefore, emphasize caution with open reduction and transphyseal fixation, and recommend limiting operative manipulation near the physis when possible [4-6,11,12]. Accordingly, when surgery is performed, the indication is typically framed as a case-specific decision to address mechanical alignment and joint function rather than a routine approach for all distal ulnar physeal injuries [5,6,8,15,17].

Recent pediatric series have also underscored that distal ulnar physeal fractures carry a meaningful risk of growth disturbance, with higher-risk patterns often including displaced and intra-articular fractures [4,6,15]. This risk profile informs clinical practice in two ways: first, it supports careful follow-up even when early radiographs appear satisfactory; and second, it reinforces the importance of minimizing additional physeal injury during treatment, whether nonoperative or operative [6,12,15]. When growth disturbance does occur, management options described in the literature range from observation in minimally symptomatic patients to later reconstructive strategies, depending on symptoms, deformity pattern, and remaining growth potential [6,12]. This highlights that the treatment course may extend beyond the initial injury period and that delayed intervention is sometimes reserved for patients who become symptomatic or develop progressive deformity over time [4,18].

In the present case, the patient sustained an isolated distal ulnar SHIV fracture and demonstrated restricted pronation and supination on initial examination, raising concern for DRUJ involvement or a mechanical impediment to forearm rotation. Initial management appropriately included closed reduction and sugar-tong splint immobilization on presentation. Operative fixation was then selected the following day based on persistent, case-specific concern for DRUJ mechanics and rotational limitation rather than as a general recommendation for distal ulnar physeal injuries. Intraoperatively, reduction was achieved and stabilized with Kirschner wire fixation, including placement of a wire across the DRUJ into the distal radius to maintain alignment. Early follow-up radiographs at two weeks and six weeks demonstrated maintained alignment and stable fixation on the available imaging.

This report contributes to the limited published experience with isolated distal ulnar SHIV fractures by providing a clearly staged radiographic sequence across multiple views (AP, lateral and oblique) from initial presentation through early postoperative follow-up. In addition, it illustrates a management pathway in which operative fixation, including temporary DRUJ transfixation, was selected based on case-specific concern for rotational limitation and DRUJ mechanics rather than as a routine approach for distal ulnar physeal injuries. Finally, the case reinforces the practical need for longitudinal surveillance after distal ulnar physeal injury, as growth-related sequelae may present in a delayed fashion even when early alignment appears maintained.

The primary limitation of this report is the incomplete long-term follow-up. Since the patient did not return beyond early postoperative imaging, long-term assessment for premature physeal closure, ulnar variance change, residual DRUJ symptoms, and functional recovery is not available. As a result, this case does not establish comparative effectiveness of operative versus nonoperative management. Instead, it provides a detailed early radiographic course and a transparent account of decision-making in the setting of rotational limitation and DRUJ concern.

## Conclusions

This case report describes a rare isolated distal ulnar SHIV fracture in an adolescent male patient with associated concern for DRUJ mechanical block/instability. Initial treatment included closed reduction and immobilization, followed by operative fixation based on restricted forearm rotation and concern for joint incongruity. Early postoperative imaging demonstrated maintained fracture alignment and DRUJ alignment. However, because the patient did not return for long-term follow-up, no conclusions can be made regarding physeal arrest prevention, long-term function, or superiority of operative management. This case supports a patient-specific approach to distal ulnar physeal injuries, with careful attention to forearm rotation, DRUJ stability, fracture alignment, and the need for ongoing follow-up to monitor for delayed growth-related complications.
